# Community-dwelling older adults’ experience after participating in intergenerational programs: A qualitative meta-synthesis

**DOI:** 10.1097/MD.0000000000042403

**Published:** 2025-05-09

**Authors:** Ying Xu, Fei Lv, Ze-Kun Bian, Wei-Yi Sun, Cai-Feng Luo

**Affiliations:** a Department of Nursing, School of Medicine, Jiangsu University, Zhenjiang, China; b Department of Medicine and Nursing, Hanlin College, Nanjing University of Chinese Medicine, Taizhou, China; c Department of Nursing, Jingjiang College, Jiangsu University, Zhenjiang, China

**Keywords:** children, experiences, intergenerational programs, meta-synthesis, older adults

## Abstract

**Background:**

Gaining a better understanding of community-dwelling older adults’ experience of participating in intergenerational programs (IGPs) is required to better care for older adults. This meta-synthesis aimed to synthesize qualitative research exploring community-dwelling older adults’ experience of participating in intergenerational programs.

**Methods:**

Seven databases (PubMed, Web of Science, Embase, CINAHL, PsycINFO, Cochrane Library, and ProQuest) were systematically searched for eligible studies exploring the experience of older adults after participating in community-based intergenerational programs. Studies were included if they: had a qualitative research method; included experiences or feelings of community-dwelling older adults to the non-family intergenerational programs; and involved intergenerational programs designed for older adults over the age of 55 and children or youth from the age of 3 to 18. The Critical Appraisal Skills Program checklist was used for the quality appraisal. Data synthesis was performed by Noblit and Hare’s methodology.

**Results:**

Thirteen articles were reviewed and synthesized. Four themes were identified. The experiences of participating in intergenerational programs among community-dwelling older adults included fostering familial connections, creating and facilitating self-worth, building community cohesion, and issues and challenges.

**Conclusion:**

The meta-synthesis proves that intergenerational programs have potential perceived benefits for older adults, but there can also be some risks and challenges if not managed well. Researchers need further research on facilitators and barriers to providing more targeted intergenerational interventions.

## 1. Introduction

To promote the health and social well-being of aging populations, intergenerational programs are being actively pursued in multiple regions to utilize the complementary resources of nonfamilial older adults and children.^[1]^ Previous quantitative studies have demonstrated the value of intergenerational programs in improving life expectancy and quality of life among older adults, enhancing the learning skills of children, promoting intergenerational relations, and addressing the challenges of limited resources in regions.^[2–4]^ Prior work has focused on quantitative studies and has less often explored the feelings and experiences of participants in the intervention. We can better understand intergenerational programs from the participants’ perspectives by understanding their perceptions of the intervention.

Communities or institutions that provide public resources and facilities to develop intentional and mutually beneficial scheduling activities for older adults and children or youth are known as intergenerational programs (IGPs).^[5]^ These programs primarily encompass 2 application scenarios: the integration of services between aged care institutions and kindergartens, and the interactions within community-based public shared spaces, differentiated by the participating groups and the content of the activities.^[6]^ Through intergenerational activities, aged care facilities assist older adults in enhancing their quality of life and reducing negative emotions,^[7]^ while the community broadens students’ life experiences while helping older adults to obtain support.^[8]^ By taking part in IGP, many older adults are able to enhance their physical activity and keep up their functional abilities.^[2,9]^ Research has shown that older adults reading books or sharing life experiences with their children in an educational setting not only enhances older adults’ intellectual performance and slows cognitive impairment to some extent,^[10]^ but also improves children’s academic achievement and behavior regulation.^[11]^ Participating in community-based activities like sculpting crafts, theatrical performances, and farm practices has also been shown to reduce loneliness and bring a perceived increase in self-worth among older adults.^[12,13]^ Intergenerational programs have also been involved in the healthcare. Structured intergenerational physical training has been found to improve cardiovascular health and obesity prevention in older adults.^[14,15]^ According to the findings of a systematic review, dementia patients may benefit from the intergenerational program by exhibiting less social isolation and finding enjoyment.^[9]^ In addition, after participating in programs, some qualitative research has indicated changes in children’s views toward older adults. For children, the programs build bridges between older adults and children, with older adults serving as grandparents, boosting children’s understanding of older adults, and reducing previously negative intergenerational perceptions.^[16,17]^

Intergenerational programs are now a crucial tool for promoting active aging in many nations because of their growing value in improving older adults’ physical health, stimulating children’s development, and strengthening community bonds. Some systematic reviews have synthesized the effectiveness of intergenerational programs on school-age children and older adults,^[2,6]^ proving that intergenerational programs can significantly reduce anxiety and increase self-perception in older adults. However, the reasons behind some older adults’ nonresponsiveness to the intervention, the high dropout rate among older individuals,^[18]^ the reduction in intergenerational interactions at follow-up, and attitude change among older adults and children cannot be captured in quantitative studies. Qualitative research, as opposed to quantitative studies, can capture more depth and nuances from a holistic perspective, giving public health and aged care a more comprehensive component.^[19]^ A current qualitative meta-synthesis study^[1]^ has examined the experiences of older adults participating in intergenerational interventions within elderly care facilities. The results indicate that these programs yield benefits while also inducing fatigue and a sense of infantilization among participants. A systematic review of mixed studies^[20]^ has similarly addressed the involvement of both older and younger individuals in intergenerational programs within nursing home settings, demonstrating that such participation can enhance well-being and social inclusion, as well as reduce age discrimination. Furthermore, research conducted by Houghton et al.^[21]^ specifically investigates the experiences and perceptions of older adults with dementia alongside younger individuals engaged in intergenerational initiatives, with both groups acknowledging that these projects foster relationships, increase interactive participation, and provide learning opportunities. The nature and implications of intergenerational program structures were also explored.

Notably, despite over 90% of older adults residing in the community,^[22]^ existing research has predominantly focused on those living in elderly care facilities. While a limited number of qualitative studies have investigated the experiences of community-dwelling older adults involved in intergenerational projects, no articles have synthesized this information. The findings of individual qualitative studies present limitations in guiding practice, leaving the overall experiences and feelings of community-dwelling older adults participating in intergenerational projects largely unexplored. Therefore, the current research aims to comprehensively synthesize qualitative studies related to intergenerational projects, with a particular emphasis on older adults’ perceptions and experiences of community involvement. This meta-synthesis seeks to address the following question: What are the experiences of community-dwelling older adults participating in intergenerational projects?

## 2. Methods

### 2.1. Study design

Meta-synthesis is the most often used method for integrating and synthesizing the results of qualitative studies by identifying overarching themes from the overall literature evidence.^[23,24]^ This systematic review and meta-synthesis were reported according to the Enhancing Transparency in Reporting the Synthesis of Qualitative Research^[25]^ and the Preferred Reporting Items for Systematic Reviews and Meta-Analyses guidelines.^[26]^

### 2.2. Search strategy and eligible criteria

Seven databases (PubMed, Web of Science, Embase, CINAHL, PsycINFO, Cochrane Library, and ProQuest Dissertations & Theses Global) were systematically searched for eligible articles written in English until October 31, 2024. Subject terms combined with text words were used as follows: (intergenerational interaction OR intergenerational activity* OR intergenerational program OR intergenerational care OR intergenerational learning) AND (elderly OR geriatric OR aged OR older adult) AND (experience or attitude or perception or view* or feel*) AND (qualitative OR phenomenology OR ethnograph* OR grounded theory OR interview*). A detailed search strategy is presented in Table 1, Supplemental Digital Content, https://links.lww.com/MD/O890. We also searched citations and references from relevant reviews to ensure the comprehensiveness of included articles. Activities for children or teenagers as well as older adults who live in the community were included. The students at college were not included because of their very different developmental traits. Articles with conference abstracts, purely quantitative design, or without full text were excluded from the review. The eligible criteria were designed by the acronym Participant, Interest in phenomenon, Context, and Study design (see Table 1).

**Table 1:** Inclusion and exclusion criteria.

	Inclusion criteria	Exclusion criteria
Population (P)	Intergenerational programs involving community-dwelling older adults over the age of 55. Intergenerational programs involve children or youth from the age of 3 to 18.	Intergenerational programs involving older adults and children are families. Intergenerational programs involving college students.
The interest in phenomenon (I)	Any experiences or feelings of community-dwelling older adults to the implementation of non-family intergenerational programs.	Focusing on other topics, such as experiences or feelings of children rather than older adults.
Context (Co)	Structured or semi-structured intergenerational programs for nonfamily community-dwelling older adults and children, including education, crafts, performances, or other creative activities conducted in the school, the community, or daycare centers.	
Study design (S)	Peer-reviewed qualitative or mixed-method studies include but are not limited to using phenomenology, grounded theory, ethnography, and other research methods.	Purely quantitative design

### 2.3. Study selection

According to the screening process and eligible criteria, 2 reviewers (Ying Xu and Fei Lv) independently screened the titles and abstracts after deleting the duplicates. For the possible studies, 2 reviewers read the full text to decide which ones met the inclusion criteria. When there was any disagreement among the reviewers, a third reviewer (B.Z.K.) would evaluate and determine the eligibility of the studies.

### 2.4. Quality appraisal

We used the Critical Appraisal Skills Programme ^[27]^ (https://www.casp-uk.net/) to appraise the quality of included studies. Researchers (Ying Xu and Fei Lv) were independently required to systematically evaluate the quality of a study with 10 items by rating “Yes,” “No” or “Can’t tell” for each item. If two-thirds of the 10 items were “Yes,” the quality level was rated as “high”; if we got “Yes” for 4 to 6 items, it was graded as “Medium” quality; if we got “No” for more than two-thirds of items, the study was considered as “low” quality. If the 2 reviewers could not come to an agreement after discussion, a third reviewer (K) would resolve the dispute.

### 2.5. Data extraction and synthesis

An Excel database (Table 2) was used by 2 reviewers (Ying Xu and Fei Lv) to obtain study characteristics independently. We notified the article’s author if the article lacked data. The extracted information included the study, country, aim, setting, participants, design, intervention, data collection, data analysis, and main results.

**Table 2:** Basic information of the included studies (n = 13).

Study & country	Aim	Setting	Participants	Study design	Interventions					Data collection & analysis
					Conductor	Traning	Frequency & duration	Size	Program	
Fees & Bradshaw, 2003,^[28]^ USA	To assess the value of the intergenerational experience in OA.	Community	77 OA (55 to over 90 years old) and 78 Yo (preschool to grade 8)	Descriptive qualitative research	A local director, OA volunteers, and a specific group of Yo	YES	Long-term program with over 55 sites; NoM.	77 OA and 78 Yo	PATH Across the Generations program	Focus groups; a thematic analysis.
Weintraub & Killian, 2007,^29]^ USA	To examine the perceptions of OA about how IGP impacted them.	Community	13 OA (65 to 90 years old)	Descriptive qualitative research	Intergenerational center and OA	NoM	Long-term program; lasted any part of a day but less than 24-hr care.	An OA day program and a Yo care center	Group programs, such as music groups, and dance groups.	Semi-structured interview; a thematic analysis.
Reisig & Fees, 2006, ^[30^A	To investigate the effects of IGP on OA.	Seven community sites	46 OA (55 to 100 years old)	Mixed-method	OA volunteers and Yo	NoM	Long-term program	954 OA volunteers and 2654 Yo	Personal Action to Health (PATH) Across the Generations	Focus groups; a thematic analysis.
Lee et al., 2021,^[31]^ South Korea, USA	To explore the experiences of OA volunteers in educational settings.	Community	23 Korean (61 to 73 years old) and 20 US (60 to 84 years old)	Phenomenology	OA volunteers	NoM.	Long-term program.	NoM.	Foster Grandparent Program in USA and Beautiful Story Grandma in Korea.	Semi-structured interview; a thematic analysis.
Kleijberg et al., 2020, ^[13]^ Sweden	To explore Yo and OA’s motivation and experiences in participation.	NoM	8 Yo (9-year-olds) and 8 OA (aged 65+) in each iteration	Action research	Research and municipal organization for culture	Yes	Two Studio DöBra iterations (2016, 2018).	8 Yo and 8 OA	A series of arts activities related to end-of-life themes.	Field notes, photographs, interviews; interpretive description.
de Souza, 2003, ^[]^ Brazil	To evaluate the IGP from the participants’ perspective.	School and community	26 OA (aged 60+) and 84 Yo(seventh and eighth grade)	Descriptive qualitative research	Two underThe synthesis of qualitative research also demonstrated how two-way intergenerational dialogue can promote intergenerational respect and mutual benefit, a theme that has also been evidenced in other studies graduate female	Yes	At least a year.	9 groups of Yo and 3 groups of OA	Reminiscence activities.	Focus groups; a thematic analysis.
Heyman & Gutheil, 2008^[34]^, USA	To understand the experiences of participants to provide insights to promote the program.	Suburban center	6 OA (age 55 to over 100), 10 Yo (aged 8+), 10 caregivers/ parents, and 10 staff	Descriptive qualitative research	the Yo, OA and staff	NoM	Small group IGP: three times a day, large group activities:monthly	About 139 children and 45 older per day	Book reading and performance.	Focus groups; open coding
Leong et al., 2022^[37]^, Singapore	To explore older people’s views on IGP.	Senior daycare center	20 OA (aged 60+) and 20 Yo (aged 13 to 16)	Descriptive qualitative research	the OA (aged 60+) and Yo	NoM	4 weeks,1.5 hours per course.	20 OA and 20 Yo	Creative and social activities such as singing, arts, and crafts.	Semi-structured interview; a thematic analysis.
Belgrave & Keown, 2018,^[17]^ USA	To examine the effects of intergenerational music collaboration on Yo and OA’s comfort and expectations.	Community	18 OA (aged 61 to 79) and 14 Yo (aged 9 to 14)	Mixed-method	the OA and Yo	Yes.	4 weeks,half a day at a time, once a week.	18 OA and 14 Yo	Intergenerational musical program, such as virtual exchanges, seminars, and performances.	Journals about open-ended questions; a content analysis.
McConnell & Naylor, 2016,^[35]^ Canada	To determine the feasibility of an intergenerational-physical-activity leadership intervention	School and community	9 OA (aged 55+) and 22 elementary school students (grade 4/5)	Mixed-method	the OA and the students	.Yes.	6 week, once a day	9 OA and 22 Yo	After-school physical activities.	Focus groups and semi-structured interviews; a thematic analysis.
Wilson et al., 2013, ^[36]^ Australia	To investigate the OA’ experiences with the program and their views about the boys.	School and community	6 older male mentors (60 to 75 years old)	Grounded theory	the OA (aged 60+)	Yes.	10 weeks,1 day at a time,once a week.	6 older male mentors and 9 boys	Shared construction project	Focus group and individual interviews; comparative approach
Highmang et al., 2023, ^[37]^ Western Australia	To study the experiences of community members in an intergenerational music group.	Community	9 OA volunteers and 8 parent participants	Descriptive qualitative research	Facilitators, OA volunteers, and parents/ carers	Yes	10 years，every week，lasting for one morning.	8 Parents/ carer,7 OA and 2 facilitators.	Music Connections Group.	Semi-structured interview; a thematic analysis.
Cohen-Mansfield & Muf,2021, ^[37]^ Israel	To investigate how various community-based IGP influence the perceived effects on both OA and Yo	Community	84 older, and 96 youngers	Mixed-method	OA, and Yo	NoM	Over a year, NoM of frequence	84 OA, and 96 Yo	IGP including art, learning and asistance OA.	questionnaire and interviews; thematic approach

IGP = intergenerational programs, NoM = Not mention, OA = Older adults, USA = United States, Yo = youth or children

We followed Noblit and Hare’s methodology^[39]^ to guide this meta-synthesis, further informed by additional updated guidance from France et al^[40]^ and Sattar et al.^[41]^ With regard to these 7 steps, meta-ethnography can be distinguished from other forms of meta-synthesis, emphasizing understanding the translation dynamics between studies, such as reciprocal or refutational relationships, and crafting a coherent argument that encapsulates the essence of the synthesized findings.^[30]^ To begin, Y.X. and W.-Y.S. thoroughly went through each of the included studies to extract metaphors from the findings, respectively, and entered these metaphors into a table to identify topically similar key metaphors. These were then organized on a matrix in theme and chronological sequence to aid in the synthesis and integration of a complete expression through a comparison of research findings within and across individual studies. In the event of a disagreement, a third reviewer (F.L.) would be brought in to help to a consensus through discussion.

## 3. Results

**Figure 1.**
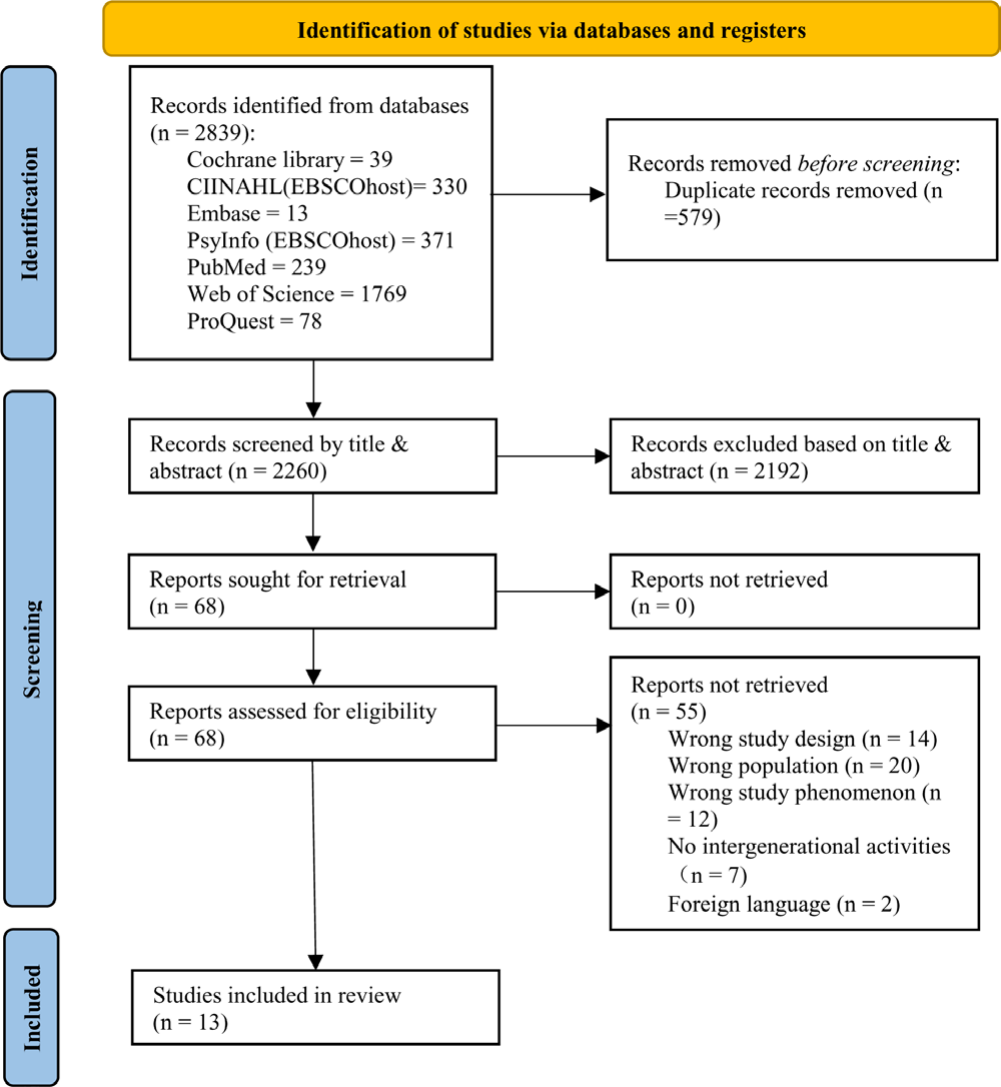
PRISMA flowchart. PRISMA =Preferred Reporting Items for Systematic Reviews and Meta-Analyses.

There were a total of 2839 possible articles found (Fig. 1). After removing 579 duplicates, 2260 articles were screened for titles and abstracts. 68 full-text articles needed to be reviewed based on the eligibility criteria, with 55 articles being excluded. In addition, one record was discovered from other sources. Finally, 13 studies met all inclusion criteria and were included in the meta-synthesis.^[13,17,31–41]^

### 3.1. Quality appraisal of the studies

All 13 studies were of good methodological quality for meeting at least 8 of 10 items (Table 3). All studies stated the study aims, qualitative methodology, study design, recruitment strategy, data collection method, data analysis, findings, and contributions. Four studies did not state the role of the researcher and the researcher-participant relationship. Four studies did not provide information on ethical approval, with 2 of them containing no discussion of any ethics in their content (neither clinical approval was stated nor any ethical issues related to the study were discussed). Finally, all 13 studies were included in the meta-synthesis.

**Table 3:** Quality appraisal of the included studies (n = 11).

Study/CASP items	1	2	3	4	5	6	7	8		10	Number of items with “Y”	Classification of Quality
Fees & Bradshaw, 2003^[28]^	Y	Y	Y	Y	Y	N	Y	Y	Y	Y	9/10	High
Weintraub & Killian, 2007^[29]^	Y	Y	Y	Y	Y	Y	N	Y	Y	Y	9/10	High
Reisig & Fees, 2006^[30]^	Y	Y	Y	Y	Y	N	Y	Y	Y	Y	9/10	High
Lee et al., 2021^[31]^	Y	N	Y	Y	Y	N	Y	Y	Y	Y	8/10	High
Kleijberg et al., 2020^[13]^	Y	Y	Y	Y	Y	Y	C	Y	Y	Y	9/10	High High
de Souza, 2003^[32]^	Y	Y	Y	C	C	C	N	Y	Y	Y	6/10	Medium
Heyman & Gutheil, 2008^[33]^	Y	Y	Y	Y	Y	Y	C	Y	Y	Y	9/10	High
Leong et al., 2022^[29]^	Y	Y	Y	Y	Y	C	Y	Y	Y	Y	9/10	High
Belgrave & 3^[17]^	Y	Y	Y	Y	Y	Y	Y	Y	Y	Y	10/10	High
McConnell & Naylor, 2016^[35]^	Y	Y	Y	Y	Y	N	Y	Y	Y	Y	9/10	High
Wilson et al., 2013^[36]^	Y	Y	Y	Y	Y	N	Y	Y	Y	Y	9/10	High
Highman et al, 2023^[37]^	Y	Y	Y	Y	Y	N	Y	Y	Y	Y	9//10	High
Cohen-Mansfield J, Muff A, 2022^[38]^	N	Y	Y	Y	Y	N	Y	Y	Y	Y	8/10	High

1 = Was there a clear statement of the aims of the research?

2 = Is a qualitative methodology appropriate?

3 = Was the research design appropriate to address the aims of the research?

4 = Was the recruitment strategy appropriate to the aims of the research?

5 = Was the data collected in a way that addressed the research issue?

6 = Has the relationship between researchers and participants been adequately considered?

7 = Have ethical issues been taken into consideration?

8 = Was the data analysis sufficiently rigorous?

9 = Is there a clear statement of findings?

10 = How valuable is the research?

CASP, Critical Appraisal Skills Programme, Y = yes; N = no; C = Can’t tell

### 3.2. Characteristics of the included studies

The included 13 studies were published from 2003 to 2023. Participants in the research featured came from a wide range of countries, including the United States,^[17,31–34,36]^ Korea,^[34]^ Sweden,^[13]^ Brazil,^[35]^ Singapore,^[37]^ Canada,^[38]^ Australia,^[39,40]^ and Israel.^[41]^ The qualitative designs of included studies were varied, 6 studies^[31,32,35–37,40]^ utilizing descriptive qualitative design, 4 studies^[17,33,38,41]^ following by mixed-method, 1 study^[34]^ following phenomenology, 1 study^[13]^ following action research approach and 1 study^[39]^ conducting grounded theory. Focus groups (n = 6), formal or semi-structured interviews (n = 8), journal or diary (n = 1), and open-ended questionnaire (n = 2) were primarily used to obtain qualitative data. Thematic analysis was the most commonly utilized data analysis method (n = 10), followed by grounded theory (n = 1), interpretative description (n = 1) and content analysis (n = 1). The sample size ranged from 6 to 84 older adults, and all of the participants have prior IGP experience. Table 2 presents the characteristics of the included studies.

### 3.3. Main findings of the meta-synthesis

Twenty-nine key metaphors were collected from the included studies (Table 4) and organized into 4 themes. Four overarching themes were revealed to be associated with older adults’ experiences of participating in an intergenerational intervention: Fostering “familial” connections; creating and facilitating self-worth; building community cohesion; and issues and challenges.

**Table 4:** Individual study metaphors related to four overarching themes.

Study	Metaphors underpinning the four themes			
	Fostering familial connections	Creating and facilitating self-worth	Building community cohesion	Issues and challenges
Fees and Bradshaw, 203^[28]^	Take on the role and responsibility of grandparents	Importance to children’s growth and development	Two-way friendship	
Weintraub and Killian, 2007^[29]^	The feeling of having a child	Being needed	Obtain peer support	
Reisig and Fees, 2006^[30]^		Physical and mental therapy	Stay connected to the community	
Lee et al., 2021^[31]^	Self-positioning grandparent	Self-worth and social presence	Sense of social affiliation	Lack of belonging
Kleijberg et al., 2020b^[13]^		Increase social engagement	Positive community atmosphere	
de Souza, 2003^[32]^		Creative and fulfilled	Provide intergenerational educational learning space	Lack of material resources and security
Heyman and Gutheil, 2008^[33]^		Emotional well-being		Low attendance
Leong et al., 2022^[34]^		Improve self-esteem and self-confidence	Two-way communication and empathy	Generation gap causes communication barriers
Belgrave and Keown, 2018^[35]^		Relaxed and pleasant	Change preconceived notions about children	
McConnell and Naylor, 2016^[35]^		A process of mutual learning	Healthy and active aging	Lack of emphasis on the intervention
Wilson et al., 2013^[36]^		A value-led mentor	Mutual respect and benefit	
Highmang et al., 2023^[37]^		A sense of connection	Community outreach	
Cohen-Mansfield & Muf, 2021^[38]^		Gaining a sense of meaning; Improved quality of life	Relationship-building	Negative emotions, tension in intergenerational relationship

#### 3.3.1. Theme 1: Fostering “familial” connections

Many older adults emphasized that the IGP helped them build new relationships by providing numerous opportunities to interact with different people. These relationships brought them a variety of unique experiences, which were categorized into 2 subthemes: mutual respect and reciprocity and self-positioning as a grandparent.

- Mutual respect and reciprocity

Older adults stated that participating in the IGP program strengthens the understanding between the 2 generations and corrects some of their preconceived notions about the younger generation. They noted that the positivity and respect demonstrated by the youth alleviated some of their initial concerns.

“*These youngsters are very respectful of us, very good. They are not affected when they see that the elderly are unkempt.”*^[37]^

“*We realized that not all young people are troublesome and they realized that not all older people are boring or quarrelsome*.”^[35]^

They acknowledged the significance of the project in fostering mutual learning, understanding, and respect. As one participant remarked, *“The integration is fantastic. We learn from them, and they learn from us. Over time, they became increasingly affectionate and friendly towards older people. It brings happiness.”*^[35]^

- Self-positioning as a grandparent

Older participants emphasized that the IGP program created a family-like atmosphere, which they deeply needed. They naturally saw themselves as grandparents, friends, or mentors to the youth, taking on the responsibility of caring for and educating them.

“*Because we are the elderly, (it is) our responsibility to instill good values in them. It is a form of care towards the youngsters and their family, their parents*.”^[37]^

“*Nowadays, generations are too divided …… I hope the children that I work with remember the warmth and love I give to them*.”^[34]^

Some older participants shared their regrets about neglecting their families in their younger years and expressed that the IGP program provided them with a second chance for intergenerational contact and to observe their family’s growth.

“*I had three daughters, and the problem was I was so busy when they were young; I didn’t have time to appreciate them. I was knocking myself out trying to get, you know, provide for the family and get going, create some kind of career uh, which makes you neglect the finer things, and you wish that you had it to do over again. Hindsight is pretty good*.”^[32]^

“*I am giving the love to other children, the love that I wanted to give to my own.*”^[34]^

#### 3.3.2. Theme 2: Creating and facilitating self-worth

Aging not only signifies a decline in physical abilities but also involves a reduction in social status. As the IGP provided opportunities for older adults to rediscover their value, they experienced a sense of creation and various benefits.

- Creative and fulfilled

Older participants emphasized that children seemed almost magical, as interacting with them always brought feelings of joy, fulfillment, a sense of being needed, and happiness.

“*I love every minute of it! Kids have a certain magic that makes my day better than teenagers in many ways*.”^[17]^

They stated that participating in the IGP was full of creativity and unpredictability, as children brought them many new experiences, such as a sense of young and an affirmation of their own life.

“*They know, you get affirmation too and that’s something that older adults are desperately needing too: recognition and affirmation*.”^[31]^

What’s more, intergenerational engagement creates unique value for older adults to recognize self-worth and maintain a social presence. They mentioned that they were often rejected and excluded from social activities due to their age, whereas the IGP provided an opportunity for social participation.

“*It just feels good that you’re in a place where you can make a difference in today’s world*.”^[34]^

“*It’s fun to be allowed to participate, to count, even though you’re old*.”^[13]^

Most older participants noted that retirement meant losing social roles and status, requiring time to adjust. They highlighted that the IGP helped them regain former roles or find new ones, fostering fulfillment and self-transcendence.

“*I was raised in a deep rural area. I knew I was good at math and wanted to be a teacher, but I couldn’t teach because I didn’t go to college. When I heard about this volunteering, I thought this is the way I can make my dream come true*.”^[34]^

- Physical and mental therapy

Older participants expressed that participating in the IGP provided both physical and psychological healing. Most participants mentioned feeling more energetic, less loneliness, and the experience of forgetting troubles while interacting with the children.

“*Because it is awful easy to just sit at home and moan and groan and feel bad and if you don’t, um, if you’ve got the kids, you forget*.”^[33]^

“*That I was not so lonely [...] it is really good for me if there is someone for whom to open my door.”“It lets me forget some of my problems*”^[41]^

Some IGPs offered intergenerational activities similar to cognitive training, and some older participants reported improvements in their attention and cognitive abilities through these activities.

“*They use the computers. Let us guess things from the screen, we have to memorize the items very fast. I can still remember the next day, our brains are very good.*”^[37]^

Moreover, some older participants noted that long-term participation in the IGP program inspired them to exercise and focus on health maintenance, driven by their desire to sustain long-term interactions with the children.

“*It keeps you young because you don’t want to let ‘em down...you want to be able to do the things with them that they want you to do*.”^[33]^

#### 3.3.3. Theme 3: Building community cohesion

The IGPs were mostly conducted within the communities where older adults lived, and as a result, it often fostered community cohesion and created a positive community atmosphere. This could be summarized into 2 subthemes: a positive community atmosphere and giving back to their communities.

- A positive community atmosphere

At the intergenerational area, older participants could freely choose to interact with children, peers, or staff. These casual interactions seemed to foster a sense of connection and belonging.

“*I thought, that was fantastic. And that’s not all, but the last time we were there, one of their mothers stayed and talked with us. [Turned out] that they had gone home and told [their parents] that they had met us*.”^[13]^

“*The devotion of the staff seems to be limitless. They’ll do anything to keep us entertained but the program itself is very important to me. There’s no one thing that I uh, am partial to, only the freedom we have in the activities, and we can just about do as we please when we get here, you know*.”^[32]^

Some older adults said that participating stimulated them to be active and helped them connect with other older participants living close by.

“*We learn many things for our own benefits from our colleagues because one needs to learn how to defend himself against illnesses.”(de Souza, 2003).*

Additionally, they shared that informal interactions with children outside the intergenerational center brought them feelings of warmth and happiness.

“*I met Matilda and Stella (children) [at the grocery store]. And they got so happy and greeted me*,”^[13]^

- Give back to their communities

Older participants had more time to engage in meaningful activities and stated that the IGP provided them with opportunities to give back to the community.

“*We see it as part of our role to reach out into the community and make contact with people and give them things to do that bring them pleasure*.”^[40]^

“*Giving back, giving more, mind you when I walked out the door 15 years ago I said no more, but 2 years later I was running, if you like, mentoring programmes!*”^[39]^

#### 3.3.4. Theme 4: Issues and challenges

Despite the advantageous effects of intergenerational interventions for individuals, families, and communities, intergenerational programs face some risks and challenges. Three subthemes emerged: generational gaps and language barriers, challenges in conducting intergenerational activities, and limitations of time and space.

- Generational gaps and language barriers

The generation gap between older adults and children was a hindrance to their communication and integration.

“*Yeah so, I think the communication is hard and that’s something we should clarify*”^[38]^

“*I am unsure about how we will collaborate with them. How will we get to know them? How long will we be with them for?*”^[17]^

Some older adults who spoke in Chinese and/or dialects experienced the communication barrier, while not all the students were proficient in them.

*“(Communication is) very difficult (with them). They don’t speak Chinese. They don’t understand the language I am saying*.”^[37]^

- Challenges in conducting intergenerational activities

The content of the intergenerational program influenced the older adults’ willingness to participate in intergenerational activities. When the activities were boring, older adults showed lower enthusiasm and interest.

“*Square ball—seems to be the most boring game in the world*.”^[38]^

Some participants mentioned that certain intergenerational activities would remind them of past regrets and negative experiences, which led to some resistance on their part.

“*I don’t want to talk about those [memories]. I think it’s really very unpleasant. I’ve seen too much*.”^[13]^

Additionally, during the implementation of intergenerational activities, older adults reported some leadership challenges, as the children seemed unwilling to cooperate with the activities.

“*There were...kids that didn’t want to participate even with their peers*.”^[36]^

- Limitations of time and space

Many participants noted that the students spent limited time at the SDCC and suggested that extending their visits or increasing their frequency would help foster stronger relationships.

“*But the time that they spent here is very short. Too short, did not get to communicate with them*.”^[37]^

“*It’s just that I have so much to do, because I have gymnastics on Saturdays and then football on Sundays […] in that case it’s another thing to add […] But otherwise I’d really like to continue with this*.”^[13]^

## 4. Discussion

Previous research on IGPs has predominantly concentrated on institutional environments. Due to the relative closure and stable composition of personnel in these settings, intergenerational interventions offer significant value in helping older adults within institutions regain social roles through interaction. However, this study shifts its focus to the experiences and perceptions of community-dwelling older adults participating in IGPs, with the aim of uncovering the potential value of these interventions within a broader social context. By employing a qualitative synthesis approach, this study identifies 4 core themes related to the experiences of community-dwelling older adults in intergenerational programs: fostering familial connections, creating and facilitating self-worth, building community cohesion, and issues and challenges. These themes provide empirical support for the effectiveness of intergenerational interventions, comprehensively reflecting the multidimensional experiences of older adults in these programs.

According to the synthesis, intergenerational interventions demonstrate the benefits of establishing connections for intergenerational groups that extend beyond familial ties, which is similar to findings from other studies.^[21]^ The lack of family connection and peer support is a significant factor in social isolation and loneliness among older adults.^[42]^ Increased family mobility and separation of older persons and their families have resulted from urbanization, increasing the danger of intergenerational isolation between older adults and children.^[43,44]^ IGPs extend the scope of intergenerational support from family ties based on blood relations to nonkin communities. Through sustained intergenerational contact, these programs cultivate unique, natural, and reciprocal “family” relationships between children and older adults, offering older individuals a distinctive “family” experience. Whereas this experience was not based on quantitative studies, highlighting the necessity of qualitative research in this field. To maximize the emotional resonance of interventions, future IGPs should include family features.

In contrast to previous meta-analytic studies on IGPs, this research reveals a distinct finding: intergenerational interventions facilitate the creation and enhancement of self-worth among community-dwelling older adults. This outcome may be attributed to earlier studies that primarily concentrated on older adults residing in institutional environments, where they often encounter more restricted social circles and daily routines. In comparison, community-dwelling older adults can engage more autonomously in intergenerational activities. Through interactions with younger generations, they rediscover their abilities and potential, which in turn enhances their sense of self-identity and social value. This was especially clear in the organized intergenerational intervention, and the review’s favorable findings offer essential recommendations for ongoing research into creative IGPs. The fact that IGPs can be beneficial in promoting older individuals’ active social engagements and improving their quality of life makes them a potentially valuable therapeutic approach.^[8]^

The synthesis of qualitative research also demonstrated how two-way intergenerational dialogue can promote intergenerational respect and mutual benefit, a theme that has also been evidenced in other studies.^[1]^ This is another issue that is not addressed in quantitative studies. The literature is clear that older adults perceive that the younger generation does not appreciate them and that their experiences go unnoticed.^[45]^ Similarly, the younger generation commonly believes that older people are incapable of comprehending their thoughts and current life situations.^[46,47]^ The evidence demonstrates that intergenerational relationships can be positive, and intergenerational understanding can be acquired through intergenerational interventions.^[48]^ This mutually beneficial program may assist older adults in establishing a sense of social affiliation while also fostering community cohesion.

However, the meta-synthesis did reveal that IGPs have some developmental issues and challenges. Similar to earlier research findings,^[1]^ the synthesis indicated that some older adults become fatigued and disinterested in activities during participation. Due to cultural variations that pose difficulties for program implementation, several older adults indicated that they had a generational gap with children and were unable to find common ground on which to speak. Intergenerational participants in each project should share a common cultural background and identity.^[49]^ There were also indications of participants’ unwillingness to cooperate and organizers’ inability to integrate older adults and children. This revealed the lack of a role for older individuals, as well as the need for organizers in assisting participants in clarifying their positions. The review was also clear that not all older individuals were happy with the activities’ substance and material security. This reflected that the interests and needs of the participants may not have been completely considered. In healthcare, it is often necessary to understand patient needs before designing the treatment plan, and IGPs should do the same. Intergenerational interventions should include participant and risk assessments as part of the planning and implementation process.

### 4.1. Strengths and limitations

Although there have been systematic reviews of the intergenerational program, this review is the first to attempt to integrate and explore the evidence of community-dwelling older adults’ experiences. Future research could look at the opinions and feelings of other relevant stakeholders about IGPs, as this study focused primarily on the experiences of community-dwelling older adults. For instance, this study only looked at English-language literature and did not do a search for gray literature, which could have resulted in some research being missed. We were unable to identify the experiences and views of older adults based on demographic characteristics due to the limited number of research studies found in this review. Finally, diverse interventions and community locations may have an impact on older individuals’ experiences. However, this provided a more varied perspective for examining older adults’ perceptions.

### 4.2. Implications for future research

The risks and obstacles associated with intergenerational program interventions are increasing as the research expands. Evidence proved that activity evaluation and staff training are now the most significant barriers to the long-term growth of IGPs in many nations.^[50]^ Researchers and community practitioners should optimize the intergenerational program. Different types of intergenerational interventions need to be developed based on the interests, abilities, and needs of older adults to avoid some undesirable effects.

While designing responsive programs for older adults, emphasis should be placed on stakeholder participatory design, also known as co-design, in program development.^[51]^ This design approach promotes active co-creation among multiple stakeholders, enhancing both the overall design outcome and its potential impact. Therefore, the design and implementation of intergenerational programs should include older adults, caregivers, family members, health professionals, and community representatives, who, as collaborators in the research, actively participate in the entire research process and share decision-making authority. Participatory design helps ensure that the program structure aligns closely with the target population’s needs and preferences, thereby enhancing both relevance and effectiveness. However, few studies have involved stakeholders in the co-design of interventions. Among the literature included in this study, only 1 explicitly indicates the use of participatory design,^[17]^ highlighting a critical area for improvement within this field.

Identifying methods to manage and evaluate the effectiveness of intergenerational interventions is also essential. There has not yet been a recognized tool created for evaluating IGPs. A set of assessment tools for use before, during, and following the implementation of activities was recently created by JARROTT and his team as part of an intergenerational evaluation toolkit.^[52]^ The Fourteen Intergenerational Best Practices Checklist can be used to assess the value of activities. It shows that if the program is carried out in accordance with the 14 requirements, the intergenerational experience and efficacy may be boosted.^[45]^ However, researchers still need to confirm the tool’s genuine validity.

The 4 themes identified in this study provide guidance for future research on the challenges of IGP implementation. Researchers need to explore the specific difficulties and limitations that IGPs encounter to develop more tailored and effective interventions. Furthermore, incorporating stakeholder insights in a co-design approach can help address real-world constraints these programs face. Communities and institutions should also provide essential support to facilitate optimal resource integration, thereby enhancing social participation and quality of life for older adults. Such a participatory approach may lead to more sustainable intergenerational programs and foster positive, lasting interactions between generations.

## 5. Conclusion

The findings of this study support the importance of IGPs in promoting active and healthy aging. Intergenerational interventions could help strengthen community cohesiveness through fostering familial connections and promoting intergenerational respect and understanding. IGPs also give older adults the chance to contribute to society. However, the synthesis also recognizes the risks and constraints of IGPs, prompting future research to look into alternative forms of interventions and include the themes raised in this study. Meanwhile, to avoid certain undesirable impacts, it is critical to consider the opinions and needs of participants when structuring the program.

## Author contributions

**Data curation:** Ying Xu, Fei Lv.

**Funding acquisition:** Ying Xu.

**Writing – original draft:** Ying Xu, Fei Lv, Ze-Kun Bian, Wei-Yi Sun.

**Formal analysis:** Fei Lv, Ze-Kun Bian, Wei-yi Sun.

**Supervision:** Cai-Feng Luo.

**Writing – review & editing:** Cai-Feng Luo.

## Supplementary Material


