# Supplementary material for: Community-dwelling older adults’ experience after participating in intergenerational programs: A qualitative meta-synthesis
Source: Medicine (Baltimore). 2025 May 9;104(19):e42403. doi: 10.1097/MD.0000000000042403 (PMC12073866; doi:10.1097/MD.0000000000042403)
Supplement: Supplementary file 1 [file medi-104-e42403-s001.pdf]

Supplementary - Table 1. Databases search strategy

Database	Search terms	Results
Pubmed		
#1	(intergenerational activity[MeSH Terms]) OR (intergenerational care[Title/Abstract] OR intergenerational interaction[Title/Abstract] OR intergenerational activit*[Title/Abstract] OR intergenerational program[Title/Abstract] OR intergenerational project OR intergenerational learning[Title/Abstract] OR intergenerational exchange[Title/Abstract]) (intergenerational care[Title/Abstract] OR intergenerational interaction[Title/Abstract] OR intergenerational activit*[Title/Abstract] OR intergenerational program[Title/Abstract] OR intergenerational project OR intergenerational learning[Title/Abstract] OR intergenerational exchange[Title/Abstract]) OR (intergenerational activity)	2,317
#2	(older people OR older adult OR senior[Title/Abstract]) OR (("Aged"[Mesh]) OR (elderly OR geriatric))	6,494,739
#3	(experience or attitude or perception or view* or feel* or opinion) OR (experience[Title/Abstract] OR perception[Title/Abstract] OR attitude[Title/Abstract] OR view*[Title/Abstract] OR feeling[Title/Abstract] OR opinion[Title/Abstract] OR perspective*[Title/Abstract])	3,437,691
#4	(qualitative or interview* or focus group) AND (qualitative[Title/Abstract] OR interview*[Title/Abstract] OR focus group[Title/Abstract] OR phenomenolog*[Title/Abstract] OR ethnograph*[Title/Abstract] OR grounded theory[Title/Abstract])	738,415
	#1 AND #2 AND #3 AND #4	239
Web of Science		
#1	TS=(intergenerational care OR intergenerational interaction OR intergenerational activity OR intergenerational program OR intergenerational learning OR intergenerational exchange) (TOPIC)	14,484
#2	TS=(elderly OR geriatric OR aged OR older adult OR older people OR senior) (TOPIC)	11,463,500
#3	TS=(experience or attitude or opinion or view* or feel* or perception or perspective*)	7,996,267
#4	TS=(qualitative or interview* or focus group or phenomenolog* or ethnograph* or grounded theory)	2,757,535
#5	#1 AND #2 AND #3 AND #4	1,769
Embase		

#1	('intergenerational care':ti,ab,kw OR 'intergenerational interaction':ti,ab,kw OR 'intergenerational activity':ti,ab,kw OR 'intergenerational program':ti,ab,kw OR 'intergenerational learning':ti,ab,kw OR 'intergenerational exchange':ti,ab,kw)	275
#2	(elderly:ti,ab,kw OR geriatric:ti,ab,kw OR aged:ti,ab,kw OR 'older adult':ti,ab,kw OR senior:ti,ab,kw)	1,322,879
#3	(qualitative:ti,ab,kw OR interview*:ti,ab,kw OR 'focus group':ti,ab,kw OR experience:ti,ab,kw OR perception:ti,ab,kw OR attitude:ti,ab,kw OR view*:ti,ab,kw OR feelings:ti,ab,kw OR opinion:ti,ab,kw OR perspective*:ti,ab,kw)	924,130
#4	(experience:ti,ab,kw OR perception:ti,ab,kw OR attitude:ti,ab,kw OR view*:ti,ab,kw OR feelings:ti,ab,kw OR opinion:ti,ab,kw OR perspective*:ti,ab,kw)	2,949,385
#5	#1 AND #2 AND #3 AND #4	13
CINAHL(EBSCO)		
#1	SU intergenerational activity OR TI (intergenerational care OR intergenerational interaction OR intergenerational activit* OR intergenerational program OR intergenerational learning OR intergenerational exchange) OR AB (intergenerational care OR intergenerational interaction OR intergenerational activity OR intergenerational program OR intergenerational learning OR intergenerational exchange)	830
#2	SU (elderly OR geriatric OR aged) OR TI (elderly OR geriatric OR aged OR older adult OR older people OR senior) OR AB (elderly OR geriatric OR aged OR older adult OR older people OR senior)	1,222,133
#3	SU (experience or attitude or opinion or view* or feel* or perception) OR TI (experience or attitude or opinion or view* or feel* or perception or perspective*) OR AB (experience or attitude or opinion or view* or feel* or perception or perspective*)	1,168,167
#4	SU (qualitative or interview* or focus group) OR TI (qualitative or interview* or focus group or phenomenolog* or ethnograph* or grounded theory) OR AB (qualitative or interview* or focus group or phenomenolog* or ethnograph* or grounded theory)	544,727
#5	#1 AND #2 AND #3 AND #4	330
PsycInfo		
#1	SU intergenerational activity OR TI (intergenerational care OR intergenerational interaction OR intergenerational activit* OR intergenerational program OR intergenerational learning OR	4,794

	intergenerational exchange) OR AB (intergenerational care OR intergenerational interaction OR intergenerational activit* OR intergenerational program OR intergenerational learning OR intergenerational exchange)	
#2	SU (elderly OR geriatric OR aged) OR TI (elderly OR geriatric OR aged OR older adult OR older people OR senior) OR AB (elderly OR geriatric OR aged OR older adult OR older people OR senior)	778,652
#3	SU (experience or attitude or opinion or view* or feel* or perception) OR TI (experience or attitude or opinion or view* or feel* or perception or perspective*) OR AB (experience or attitude or opinion or view* or feel* or perception or perspective*)	2,095,038
#4	SU (qualitative or interview* or focus group) OR TI (qualitative or interview* or focus group or phenomenolog* or ethnograph* or grounded theory) OR AB (qualitative or interview* or focus group or phenomenolog* or ethnograph* or grounded theory)	617,119
#5	#1 AND #2 AND #3 AND #4	371
Cochrane Library		
#1	TITLE-ABS-KEY (intergenerational care OR intergenerational interaction OR intergenerational activity OR intergenerational program OR intergenerational learning OR intergenerational exchange)	1
#2	TITLE-ABS-KEY (elderly OR geriatric OR aged OR older adult OR older people OR senior)	3929
#3	TITLE-ABS-KEY (experience or attitude or opinion or view* or feel* or perception or perspective*)	2213
#4	TITLE-ABS-KEY (qualitative or interview* or focus group or phenomenolog* or ethnograph* or grounded theory)	1353
#5	#1 AND #2 AND #3 AND #4	39
ProQuest		
#1	su(intergenerational activity) OR ti(intergenerational care OR intergenerational interaction OR intergenerational activit* OR intergenerational program OR intergenerational learning OR intergenerational exchange) OR ab(intergenerational care OR intergenerational interaction OR intergenerational activit* OR intergenerational program OR intergenerational learning OR intergenerational exchange)	1728
#2	mainsubject(elderly OR geriatric OR aged) OR ti(older people OR older adult OR senior) OR ab(older people OR older adult OR senior)	105,292

#3	mainsubject(experience or attitude or perception or view* or feel* or opinion) OR ti(experience or attitude or opinion or view* or feel* or perception or perspective*) OR ab(experience or attitude or opinion or view* or feel* or perception or perspective*)	1,095,062
#4	mainsubject(qualitative or interview* or focus group) OR ti(qualitative or interview* or focus group or phenomenolog* or ethnograph* or grounded theory) OR ab(qualitative or interview* or focus group or phenomenolog* or ethnograph* or grounded theory)	419,287
#5	#1 AND #2 AND #3 AND #4	78
Total databases		2844
